# Three-to-One Internal Resonance in MEMS Arch Resonators

**DOI:** 10.3390/s19081888

**Published:** 2019-04-20

**Authors:** Ze Wang, Jianting Ren

**Affiliations:** School of Mechanics, Civil Engineering and Architecture, Northwestern Polytechnical University, Xi’an 710129, China; wangze0127@mail.nwpu.edu.cn

**Keywords:** MEMS arch resonators, nonlinear dynamics, three-to-one internal resonance, Hopf bifurcation

## Abstract

We present an investigation of the nonlinear dynamics of a microelectromechanical system (MEMS) arch subjected to a combination of AC and DC loadings in the presence of three-to-one internal resonance. The axial force resulting from the residual stress or temperature variation is considered in the governing equation of motion. The method of multiple scales is used to solve the governing equation. A four first-order ordinary differential equation describing the modulation of the amplitudes and phase angles is obtained. The equilibrium solution and its stability of the modulation equations are determined. Moreover, we also obtain the reduced-order model (ROM) of the MEMS arch employing the Galerkin scheme. The dynamic response is presented in the form of time traces, Fourier spectrum, phase-plane portrait, and Poincare sections. The results show that when there is an internal resonance, the energy transfer occurs between the first and third modes. In addition, the response of the MEMS arch presents abundant dynamic behaviors, such as Hopf bifurcation and quasiperiodic motions.

## 1. Introduction

Microelectromechanical system (MEMS) devices are widely used in various engineering applications due to their low energy consumption, high sensitivities, and small size. Examples of these include resonators [1], bio-sensors [2], actuators [3], airbag accelerometers [4], and so on. In the design of these MEMS devices, various structures are used, such as fixed-fixed microbeams [5], cantilever microbeams [6], H-shaped microbeams [7], double microbeams [8], microplates [9] and so on. Due to their attractive features, arch structures have attracted a growing amount of attention from the microelectromechanical system community. For example, it has been recently reported that arch structures are used as mechanical memories [10] and logic devices [11]. However, because of the nonlinear nature aroused by its own initial curvature, mid-plane stretching, and electric load, MEMS arches can present abundant nonlinear dynamic behaviors.

There has been much theoretical research carried out on the nonlinear dynamics of MEMS arches. For example, Ouakad et al. [12] investigated the primary resonance of the first natural frequency of a MEMS arch using the method of multiple scales. They found that the arch showed softening behavior. Younis et al. [13] proposed a reduced-order model of a MEMS arch based on the Galerkin method and then examined its primary resonance and subharmonic resonance. Krylov et al. [14], using same technique, investigated the pull-in and snap-through instability of MEMS arches. Farokhi et al. [15] constructed a high-dimensional reduced-order Galerkin model and then studied the effect of various system parameters on pull-in instabilities. Laura et al. [16,17] explored the global dynamic behavior of MEMS arches. In addition, many experimental studies on the dynamics of MEMS arch have been published. For example, Ahmad et al. [18] experimentally examined the primary resonance of the first natural frequency of an MEMS arch. Nonlinear phenomena such as jumps, hysteresis, and softening behaviors were observed. Laura et al. [19] detected super harmonic resonances in frequency sweeps experiments. Younis et al. [20] investigated the response of a MEMS arch under a large AC harmonic load experimentally. They found that the dynamic snap-through and dynamic pull-in can be induced for some specific parameters. Zhang et al. [21] examined the snap-through instability and pull-in instability of a MEMS arch with different configurations. Hajjaj et al. [22] examined the effect of initial shapes, arc, and cosine wave on the dynamic behaviors of MEMS arch resonators. However, it should be noted that the above literature only covers single-mode dynamics. Little attention had been devoted to the dynamics of multi-modes.

Recently, modal interaction has been highly emphasized by researchers. Abdallah et al. [23] experimentally studied an arch resonator in the presence of internal resonance. The results showed that the first and third modes coupled with each other. Hajjaj et al. [24] experimentally investigated an electrothermally tuned and electrostatically actuated arch resonator. The results showed that one-to-one internal resonance between the first and third modes can be activated for some specific parameters. Samanta et al. [25] experimentally detected three different internal resonances in a few-layer MoS_2_ resonator. Vyas et al. [26] designed a T-beam nano-resonator using its nonlinear modal interaction due to 1:2 internal resonance. Damian et al. [27] and Antonio et al. [28] demonstrated that for a MEMS arch clamped at both ends the internal resonance can reduce the frequency-amplitude interdependence and stabilized frequency. In addition, analytical research concerning nonlinear modal interaction has also been performed. Younis and Nayfeh [29] explored the possibility of modal interactions between the first and second modes for a MEMS resonator via the method of multiple scales. They found that the first two modes are uncoupled and that energy transfer cannot occur between the involved modes. Pourkiaee et al. [30] examined the nonlinear modal interaction of a doubly clamped piezoelectric MEMS arch. They found that the interaction between the first and third mode can be activated due to the three-to-one internal resonance.

It can be noted that analytical studies on the modal interaction in MEMS arches are scarce. Therefore, this investigation aims to thoroughly study the dynamics of a MEMS arch in the presence of three-to-one internal resonance. To accomplish this, the multiple scale method is used to solve the governing equation of motion. Frequency response curves and force response curves are obtained. In addition, a reduced-order model, using the Galerkin scheme, is derived. The dynamics of the MEMS arch is investigated with respect to the time history, phase-plane portrait, Poincare sections, and Fourier spectrum.

## 2. Problem Formulation

**Figure 1:**
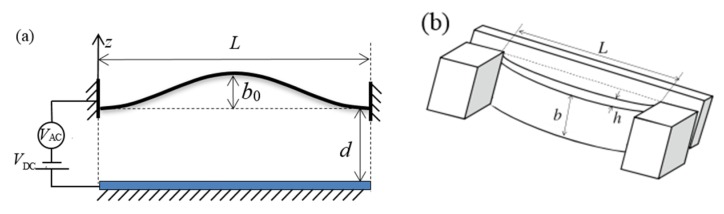
(**a**) Schematic representation of a microelectromechanical system (MEMS) arch resonator and (**b**) 3D schematic of the arch.

Figure 1 shows a MEMS arch resonator with electrostatic actuation which is composed of a deformable top electrode and a stationary bottom electrode. The deformable electrode is actuated by a distributed electrostatic force provided by the stationary electrode. The deformable electrode is modeled as a fixed-fixed arch subjected to a combined AC and DC voltage load. The initial shape of the arch is described by the function *w*_0_(*x*) = 0.5*b*_0_(1 − cos2π*x*), where *b*_0_ is the initial rise.

In the present study, the equation of motion was obtained by assuming that: (1) the arch is shallow, i.e., d*w*_0_/d*x* << 1, and, hence, the parallel-plate assumption is valid; (2) the Euler-Bernoulli beam theory may be used, neglecting the effect of shear and rotary inertia; (3) the simplest viscous damping model may be adopted to model the dissipative mechanisms of the resonator.

Therefore, the equation of motion governing the transverse deflection *w*(*x*,*t*) of the arch with length *L*, width *b*, and thickness *h* is expressed as [12,31]

(1) $$\begin{array}{l} \rho A\frac{\partial^{2}w}{\partial t^{2}}+c\frac{\partial w}{\partial t}+EI\frac{\partial^{4}w}{\partial x^{4}}+P\left(\frac{\partial^{2}w}{\partial x^{2}}+\frac{d^{2}w_{0}}{dx^{2}}\right)\\ \;-\frac{EA}{2L}\left(\frac{\partial^{2}w}{\partial x^{2}}+\frac{d^{2}w_{0}}{dx^{2}}\right)\int_{0}^{L}\left[{\left(\frac{\partial w}{\partial x}\right)}^{2}+2\frac{\partial w}{\partial x}\frac{dw_{0}}{dx}\right]dx=-\frac{\varepsilon b{\left[V_{DC}+V_{AC}\cos\left(\Omega t\right)\right]}^{2}}{2{\left(d+w_{0}+w\right)}^{2}} \end{array}$$

and the boundary condition can be expressed as

(2) $$w\left(0,t\right)=0,\;\frac{\partial w\left(0,t\right)}{\partial x}=0,\;w\left(L,t\right)=0,\;\frac{\partial w\left(L,t\right)}{\partial x}=0$$

where *E* is Young’s modulus, *G* is the shear modulus, *c* is the viscous damping coefficient, *ρ* is the material density, *d* is the gap between electrodes, *A* is the cross-sectional area, *I* is the moment of inertia, *l* is the material length scale parameter, *P* is the axial load due to residual stress, and *ε* is the dielectric constant of the air.

The governing equation can be transformed to the dimensionless forms by introducing the following parameters.

$$w=w/d,\;x=x/L,\;T=\sqrt{EI/\rho AL^{4}}$$

The governing equation and the boundary condition can be cast into the dimensionless form

(3) $$\begin{array}{r} \ddot{w}+2c\dot{w}+w^{iv}+P\left(w^{\prime \prime }+{w^{\prime \prime }}_{0}\right)-\alpha_{1}\left(w^{\prime \prime }+{w^{\prime \prime }}_{0}\right)\int_{0}^{1}\left({w^{\prime }}^{2}+2w^{\prime }w_{0}^{\prime }\right)dx\\ =-\frac{\alpha_{2}{\left(V_{DC}+V_{AC}\cos\left(\Omega t\right)\right)}^{2}}{{\left(1+w_{0}+w\right)}^{2}} \end{array}$$

(4) $$w\left(0,t\right)=0,\;\frac{\partial w\left(0,t\right)}{\partial x}=0,\;w\left(1,t\right)=0,\;\frac{\partial w\left(1,t\right)}{\partial x}=0$$

where the overdot indicates the derivative with respect to *t*, the prime indicates the derivative with respect to *x*, and

(5) $$\alpha_{1}=6{\left(\frac{d}{h}\right)}^{2},2c=\frac{cL^{4}}{EI}\sqrt{\frac{EI}{\rho AL^{4}}},\alpha_{2}=\frac{6\varepsilon L^{4}}{Eh^{3}d^{3}},P=\frac{PL^{2}}{EI}$$

## 3. Method of Analysis

In this section we used the method of multiple scales to solve the response of the arch under a combined AC and DC voltage load. To this end, we introduced the time scales *T*_0_ = *t*, *T*_1_ = *εt*, and *T*_2_ = *ε*^2^*t*, where *ε* is a small dimensionless bookkeeping parameter. Moreover, in order for nonlinearity to balance the effects of the damping *c* and the excitation *V_AC_*, we rescaled them as *ε*^2^*c* and *ε*^3^*V_AC_*. Hence, we sought a solution for the governing Equation (3) in the form

(6) $$w\left(x,t;\varepsilon \right)=w_{s}\left(x\right)+\upsilon \left(x,t\right)=w_{s}\left(x\right)+\sum_{i=1}^{3}\varepsilon^{i}\upsilon_{i}\left(x,T_{0},T_{2}\right)\left(a\right)$$

where *w_s_*(*x*) denotes the static component of the arch due to the DC voltage and *υ*(*x*,*t*) denotes the dynamic component due to the AC voltage. Substituting Equation (6) into Equations (3) and (4) and equating coefficients of like powers of *ε*, we obtained

(7) $$O\left(\varepsilon^{0}\right):w_{s}^{iv}+\left[P-\alpha_{1}\int_{0}^{1}\left({w^{\prime }}_{s}^{2}+2w_{s}^{\prime }w_{0}^{\prime }\right)dx\right]\left(w_{s}^{\prime \prime }+w_{0}^{\prime \prime }\right)+\frac{\alpha_{2}V_{DC}^{2}}{{\left(1+w_{0}+w_{s}\right)}^{2}}=0$$

(8) $$\begin{array}{ll} O\left(\varepsilon^{1}\right):L\left(\upsilon_{1}\right)= & D_{0}^{2}\upsilon_{1}+\upsilon_{1}^{iv}+\left(P-\alpha_{1}\int_{0}^{1}\left({w^{\prime }}_{s}^{2}+2w_{s}^{\prime }w_{0}^{\prime }\right)dx\right){\upsilon^{\prime \prime }}_{1}\\ & -2\alpha_{1}\left(w_{s}^{\prime \prime }+w_{0}^{\prime \prime }\right)\int_{0}^{1}\left(w_{s}^{\prime }+w_{0}^{\prime }\right)\upsilon_{1}^{\prime }dx-\frac{2\alpha_{2}V_{DC}^{2}}{{\left(1+w_{0}+w_{s}\right)}^{3}}\upsilon_{1}=0 \end{array}$$

(9) $$O\left(\varepsilon^{2}\right):L\left(\upsilon_{2}\right)=\alpha_{1}\left(w_{s}^{\prime \prime }+w_{0}^{\prime \prime }\right)\int_{0}^{1}{\upsilon^{\prime }}_{1}^{2}dx+2\alpha_{1}\upsilon_{1}^{\prime \prime }\int_{0}^{1}\left(w_{s}^{\prime }+w_{0}^{\prime }\right)\upsilon_{1}^{\prime }dx-\frac{3\alpha_{2}V_{DC}^{2}}{{\left(1+w_{0}+w_{s}\right)}^{4}}\upsilon_{1}^{2}$$

(10) $$\begin{array}{ll} O\left(\varepsilon^{3}\right):L\left(\upsilon_{3}\right)= & -2D_{0}D_{2}\upsilon_{1}-2cD_{0}\upsilon_{1}+2\alpha_{1}\left(w_{s}^{\prime \prime }+w_{0}^{\prime \prime }\right)\int_{0}^{1}\upsilon_{1}^{\prime }\upsilon_{2}^{\prime }dx\\ & +2\alpha_{1}\upsilon_{1}^{\prime }\int_{0}^{1}\left(w_{s}^{\prime }+w_{0}^{\prime }\right)\upsilon_{2}^{\prime }dx+\alpha_{1}\upsilon_{1}^{\prime }\int_{0}^{1}{\upsilon^{\prime }}_{1}^{2}dx+2\alpha_{1}\upsilon_{2}^{\prime }\int_{0}^{1}\left(w_{s}^{\prime }+w_{0}^{\prime }\right)\upsilon_{1}^{\prime }dx\\ & -\frac{6\alpha_{2}V_{DC}^{2}}{{\left(1+w_{0}+w_{s}\right)}^{4}}\upsilon_{1}\upsilon_{2}+\frac{4\alpha_{2}V_{DC}^{2}}{{\left(1+w_{0}+w_{s}\right)}^{5}}\upsilon_{1}^{3}-\frac{2\alpha_{2}V_{DC}V_{AC}\cos\left(\Omega t\right)}{{\left(1+w_{0}+w_{s}\right)}^{2}} \end{array}$$

where *D_n_* ≡ ∂/∂*T_n_*. The boundary conditions for all orders are.

(11) $$\upsilon_{i}=0\;\mathrm{and}\;\upsilon_{i}^{\prime }=0\;\mathrm{at}\;x=\;0\;\mathrm{and}\;x=\;1\;\mathrm{for}\;i=1,2,3$$

**Figure 2:**
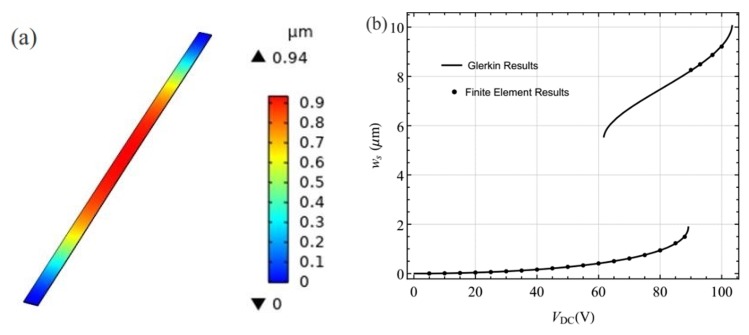
(**a**) The transverse deflection obtained using the software COMSOL when *V*_DC_ = 80 V; (**b**) variations of the midpoint static deflection with the DC voltage.

One can note that Equation (7) is the static deflection problem of the arch under the DC voltage. As a case study, we considered a MEMS arch resonator made of silicon. Its geometric and material parameters were *L* = 1000 μm, *h* = 2.4 μm, *b* = 30 μm, *d* = 10.1 μm, *b*_0_ = 3.6 μm, *P* = 0 N, *E* = 167.8 GPa, and *ρ* = 2320 kg/m^3^ [32]. Here, the Galerkin method was employed to solve Equation (7) [12]. Figure 2b shows variation of the midpoint static deflection of the arch with the DC voltage (*V*_DC_). To validate the results using the Galerkin method, the finite element method (FEM) was also employed to simulate the static deformation of the MEMS arch. It can be noted from Figure 2b that the results via the two methods are in agreement. Here, the results of the finite element method were obtained by using the software COMSOL, as shown in Figure 2a.

Equations (8)–(11) govern the dynamic response around the static configuration of the arch. Equation (8) is the linear eigenvalue problem of the arch. The solution of Equation (8) can be expressed as

(12) $$\upsilon_{1}\left(x,T_{0},T_{2}\right)=\sum_{m=1}^{\infty }A_{m}\left(T_{2}\right)\phi_{m}\left(x\right)e^{i\omega_{m}T_{0}}+cc$$

where *A_m_*(*T*_2_) denotes the complex function to be determined, *ω_m_* is *n*th natural frequency of the system, *ϕ_m_*(*x*) is the mode function, and *cc* indicates the complex conjugate of the preceding terms.

**Figure 3:**
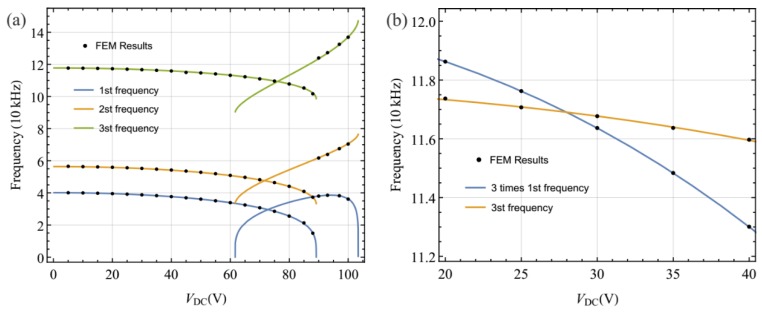
(**a**) Variation of the first three natural frequencies with DC voltage; (**b**) variations of three times the first natural frequency and the third natural frequency with DC voltage. Legend: FEM, finite element method.

Much literature has been published on the linear eigenvalue problem of the arch. For more details of the solution methodology, refer to works [12,29]. Here, Figure 3a shows variation of the first three natural frequencies with the DC voltage and Figure 3b shows variations of three times the first natural frequency and the third natural frequency with the DC voltage. In these figures, the results obtained by FEM are given. It is found that the third natural frequency is approximately equal to three times the first natural frequency, being near *V_DC_* = 27.95. Hence, there may be a three-to-one internal resonance for a specific range of parameters. The internal resonance is perfectly tuned when *V_DC_* ≈ 27.95. In the present investigation, we explored the dynamics of the system in the presence of three-to-one internal resonance between the first and third modes. Here, we assumed that neither of these two modes was involved in an internal resonance with other modes. Due to the presence of damping and internal resonance, only the first and third modes contribute to the long-time dynamic response. As a result, Equation (8) can be expressed as

(13) $$\upsilon_{1}\left(x,T_{0},T_{2}\right)=A_{1}\phi_{1}\left(x\right)e^{i\omega_{1}T_{0}}+A_{3}\phi_{3}\left(x\right)e^{i\omega_{3}T_{0}}+cc$$

Next, we offer a detailed investigation on the dynamic of the MEMS arch resonator in the presence of the internal resonance between the first and third modes.

## 4. Primary Resonance of the First Mode

To describe the nearness of the *ω*_3_ to 3*ω*_1_ and the excitation frequency Ω to *ω*_1_, we let

(14) $$\omega_{3}=3\omega_{1}+\varepsilon^{2}\sigma_{1}\;\;\Omega =\omega_{1}+\varepsilon^{2}\sigma_{2}$$

where *σ*_1_ and *σ*_2_ are detuning parameters.

Substituting Equation (13) into Equation (9) yields

(15) $$\begin{array}{ll} L\left(\upsilon_{2}\right) & =A_{1}^{2}h_{11}e^{i2\omega_{1}T_{0}}+A_{3}^{2}h_{13}e^{i2\omega_{3}T_{0}}+A_{1}{\bar{A}}_{1}h_{11}+A_{3}{\bar{A}}_{3}h_{13}\\ & +\left(A_{1}A_{3}e^{i\left(\omega_{3}+\omega_{1}\right)T_{0}}+A_{3}{\bar{A}}_{1}e^{i\left(-\omega_{1}+\omega_{3}\right)T_{0}}\right)H_{13}+cc \end{array}$$

where

$$h_{1i}=\alpha_{1}\left(w_{s}^{\prime \prime }\int_{0}^{1}{\phi^{\prime }}_{i}^{2}dx+2\phi_{i}^{\prime \prime }\int_{0}^{1}w_{s}^{\prime }\phi_{i}^{\prime }dx\right)-\frac{3\alpha_{2}V_{DC}^{2}}{{\left(1+w_{s}\right)}^{4}}\phi_{i}^{2}\;i=1,3\;H_{13}=2\alpha_{1}\left(w_{s}^{\prime \prime }\int_{0}^{1}\phi_{1}^{\prime }\phi_{3}^{\prime }dx+\phi_{3}^{\prime \prime }\int_{0}^{1}w_{s}^{\prime }\phi_{3}^{\prime }dx+\phi_{3}^{\prime \prime }\int_{0}^{1}w_{s}^{\prime }\phi_{1}^{\prime }dx\right)-\frac{6\alpha_{2}V_{DC}^{2}}{{\left(1+w_{s}\right)}^{4}}\phi_{1}\phi_{3}$$

A particular solution of Equation (9) can be expressed as

(16) $$\begin{array}{ll} \upsilon_{2}\left(x,T_{0},T_{2}\right)= & \psi_{11}\left(x\right)A_{1}^{2}\left(T_{2}\right)e^{2i\omega_{1}T_{0}}+\psi_{13}\left(x\right)A_{3}^{2}\left(T_{2}\right)e^{2i\omega_{3}T_{0}}+\\ & \psi_{3}\left(x\right)A_{3}\left(T_{2}\right)A_{1}\left(T_{2}\right)e^{i\left(\omega_{1}+\omega_{3}\right)T_{0}}+\psi_{4}\left(x\right)A_{3}\left(T_{2}\right){\bar{A}}_{1}\left(T_{2}\right)e^{i\left(\omega_{3}-\omega_{1}\right)T_{0}}+\\ & \psi_{33}\left(x\right)A_{3}\left(T_{2}\right){\bar{A}}_{3}\left(T_{2}\right)+\psi_{31}\left(x\right)A_{1}\left(T_{2}\right){\bar{A}}_{1}\left(T_{2}\right)+cc \end{array}$$

The *ψ*_ij_ are solutions of the following boundary-value problem:

(17) $$M\left[\psi_{1i}\left(x\right),2\omega_{i}\right]=h_{1i}\left(x\right)\;i=1,3$$

(18) $$M\left[\psi_{3}\left(x\right),\omega_{1}+\omega_{3}\right]=H_{12}\left(x\right)$$

(19) $$M\left[\psi_{4}\left(x\right),\omega_{3}-\omega_{1}\right]=H_{12}\left(x\right)$$

(20) $$M\left[\psi_{2i}\left(x\right),0\right]=h_{1i}\left(x\right)\;i=1,3$$

This has the boundary conditions

(21) $$\psi_{ij}=0\;\mathrm{and}\;\psi_{ij}^{\prime }=0\;\mathrm{at}\;x\;=\;0,\;1$$

where the operator M is defined as the following equation:

(22) $$\begin{array}{r} M\left(\psi ,\omega \right)=k_{1}\psi^{iv}-\omega^{2}\psi +\left[P-\alpha_{1}\int_{0}^{1}\left({w^{\prime }}_{s}^{2}-{w^{\prime }}_{0}^{2}\right)dx\right]\psi^{\prime \prime }\\ -2\alpha_{1}w_{s}^{\prime \prime }\int_{0}^{1}w_{s}^{\prime }\psi^{\prime }dx-\frac{2\alpha_{2}V_{DC}^{2}}{{\left(1+w_{s}\right)}^{3}}\psi \end{array}$$

Substituting Equations (13) and (16) into Equation (10) and considering Equation (14), we obtained

(23) $$\begin{array}{ll} L\left(\upsilon_{3}\right)= & \left[-2i\omega_{1}\left({\dot{A}}_{1}+cA_{1}\right)\phi_{1}+\chi_{11}\left(x\right)A_{1}^{2}{\bar{A}}_{1}+\chi_{13}\left(x\right)A_{1}A_{3}{\bar{A}}_{3}\right]e^{i\omega_{1}T_{0}}+\\ & \left[-2i\omega_{3}\left({\dot{A}}_{3}+cA_{3}\right)\phi_{3}+\chi_{3}\left(x\right)A_{1}^{2}{\bar{A}}_{1}+\chi_{31}\left(x\right)A_{3}A_{1}{\bar{A}}_{1}\right]e^{i\omega_{3}T_{0}}+\\ & \chi_{5}\left(x\right)A_{1}^{3}e^{3i\omega_{1}T_{0}}+\chi_{6}\left(x\right)A_{3}{\bar{A}}_{1}^{2}e^{i\left(\omega_{3}-2\omega_{1}\right)T_{0}}+F\left(x\right)e^{i\omega_{1}T_{0}}e^{i\sigma_{2}T_{2}}+cc+NST \end{array}$$

where the overdot indicates the differential with respect to *T*_2_, *NST* denotes terms that do not produce secular effects, and the functions χ’s and *F*(*x*) are defined in Appendix A.

Due to the fact that the homogeneous part of Equation (23) has a nontrivial solution, Equation (23) has a nontrivial solution only if the solvability conditions are satisfied. Because this problem is self-adjoint, it can be demanded that the right hand of Equation (23) be orthogonal to *ϕ*_m_(*x*)exp(−*iω*_m_*T*_0_) and *ϕ*_n_(*x*)exp(−*iω*_n_*T*_0_). Performing these manipulations, we were able to obtain the modulation equations

(24) $$\begin{array}{l} 2i\omega_{1}\left({\dot{A}}_{1}+cA_{1}\right)=8S_{11}A_{1}^{2}{\bar{A}}_{1}+8S_{13}A_{1}A_{3}{\bar{A}}_{3}+8\Lambda_{1}A_{3}{\bar{A}}_{1}^{2}e^{i\sigma_{1}T_{2}}+f_{1}e^{i\sigma_{2}T_{2}}\\ 2i\omega_{3}\left({\dot{A}}_{3}+cA_{3}\right)=8S_{33}A_{3}^{2}{\bar{A}}_{3}+8S_{31}A_{3}A_{1}{\bar{A}}_{1}+8\Lambda_{3}A_{1}^{3}e^{-i\sigma_{1}T_{2}} \end{array}$$

where *f*_1_, Λ*_i_*, and *S_ij_* (*i*,*j* = 1,3) are given in Appendix. *f*_1_ is the projection of the excitation force onto the first mode. *S_ij_* are nonlinear coefficients due to electric and geometric sources and the Λ*_i_* are the nonlinear interaction coefficients between the first and third modes. If the nonlinear interaction coefficients Λ*_i_* are identically zero, the involving modes are orthogonal in the nonlinear sense and there is no potential for energy transfer between them.

Next, we introduced the Cartesian transformation

(25) $$A_{i}=\frac{1}{2}\left(p_{i}+iq_{i}\right)e^{is_{i}}\;i=1,3$$

into Equation (24), separated the obtained results into those real and imaginary, and yielded

(26) $$\begin{array}{ll} q_{1}^{\prime }= & -\sigma_{2}p_{1}-cq_{1}-S_{11}\left(p_{1}^{3}+p_{1}q_{1}^{2}\right)-S_{13}\left(p_{1}p_{3}^{2}+p_{1}q_{3}^{2}\right)\\ & -\Lambda_{1}\left(p_{1}^{2}p_{3}-p_{3}q_{1}^{2}+2p_{1}q_{1}q_{3}\right)-f_{1} \end{array}$$

(27) $$\begin{array}{ll} p_{1}^{\prime }= & \sigma_{2}q_{1}-cp_{1}+S_{11}\left(p_{1}^{2}q_{1}+q_{1}^{3}\right)+S_{13}\left(p_{3}^{2}q_{1}+q_{1}q_{3}^{2}\right)\\ & +\Lambda_{1}\left(p_{1}^{2}q_{3}-q_{1}^{2}q_{3}-2p_{1}p_{3}q_{1}\right) \end{array}$$

(28) $$q_{3}^{\prime }=-s_{2}p_{3}-cq_{3}-S_{33}\left(p_{3}^{3}+p_{3}q_{3}^{2}\right)-S_{31}\left(p_{1}^{2}p_{3}+p_{3}q_{1}^{2}\right)-\Lambda_{3}\left(p_{1}^{3}-3p_{1}q_{1}^{2}\right)$$

(29) $$p_{3}^{\prime }=s_{2}q_{3}-cp_{3}+S_{33}\left(p_{3}^{2}q_{3}+q_{3}^{3}\right)+S_{31}\left(p_{1}^{2}q_{3}+q_{1}^{2}q_{3}\right)+\Lambda_{3}\left(3p_{1}^{2}q_{1}-q_{1}^{3}\right)$$

where *s*_1_ = *σ*_2_ and *s*_2_ = 3*σ*_2_ − *σ*_1_. The equilibrium solution of the system was obtained by setting $p_{1}^{\prime }=p_{3}^{\prime }=q_{1}^{\prime }=q_{3}^{\prime }=0$. Then, the resulting algebraic equation was numerically solved and the eigenvalue of the corresponding Jacobian matrix found; these were used for examining the stability of the equilibrium solution.

(30) $$a_{1}=\sqrt{p_{1}^{2}+q_{1}^{2}}\;a_{3}=\sqrt{p_{3}^{2}+q_{3}^{2}}$$

where *a*_1_ and *a*_3_ are the amplitudes of the first and second modes, respectively.

### Equilibrium Solutions and Their Stability

In the present section, numerical examples are shown to reveal characteristics of response. We consider again the MEMS arch resonator of Section 3. Here, we chose a DC voltage of 23.80, where a three-to-one internal resonance occurs, as shown in Section 3. The AC voltage was chosen to be 0.60. The corresponding dimensionless values were *α*_1_ = 106.3 and *α*_2_ = 0.023. The damping coefficient *c* was equal to 0.01. The first and third dimensionless natural frequencies were *ω*_1_ = 42.9127 and *ω*_3_ = 127.9320. These parameters were selected as fixed parameters, if no others were assigned.

**Figure 4:**
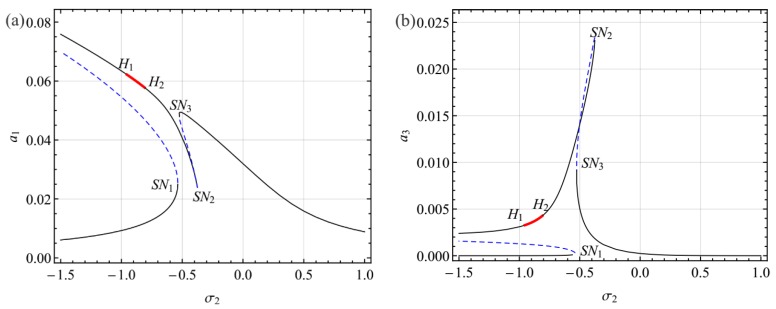
Frequency response curves for the first (**a**) and third (**b**) modes for *V*_DC_ = 23.80, *σ*_1_ = −0.8059, and *V*_AC_ = 0.6.

Figure 4 shows the frequency response curves for the first and third modes as functions of the detuning parameter *σ*_2_ near the primary resonance of the first mode. In these figures, the solid black lines denote the stable solution, the dashed blue lines denote the unstable solution, and the thick red solid lines denote unstable foci.

It follows from Figure 4 that the curves bent to the left, indicating softening spring type behavior. As *σ*_2_ increased from a small value of *σ*_2_, where two stable equilibrium solutions coexisted, the amplitude for smaller equilibrium solutions grew all the way until a saddle node bifurcation occurred at *SN*_1_. Increasing *σ*_2_ beyond *SN*_1_, the response jumped to the other equilibrium solution. For the larger equilibrium solutions, the amplitude of the first mode decreased all the way, whereas the amplitude of the second mode decreased first and then grew until a saddle node bifurcation occurred at *SN*_2_. This phenomenon indicates that the energy was transferred from the first mode to the second mode. Moreover, as *σ*_2_ increased from a small value, the response lost stability via a Hopf bifurcation at *H*_1_ and then regained stability via a reverse Hopf bifurcation at *H*_2_. It is worth mentioning that the response of the system between the two Hopf bifurcations (*H*_1_ and *H*_2_) may present rich nonlinear dynamic behaviors which can be examined by a numerical method. To reveal the characteristics of the response in this region, the Galerkin truncation scheme was employed, which the dynamic solutions presented in Section 5.

**Figure 5:**
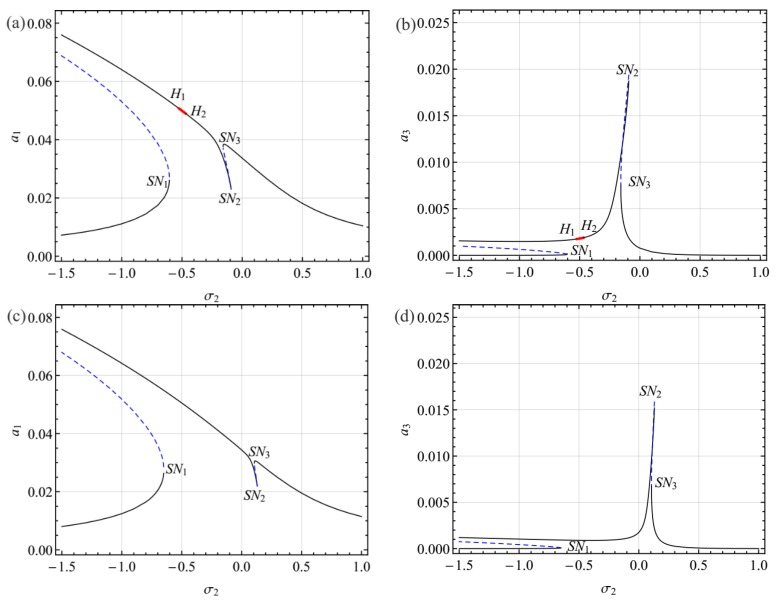
Frequency response curves for the first and third modes: (**a**,**b**) *V*_DC_ = 28.00, *σ*_1_ = 0.005; (**c**,**d**) *V*_DC_ = 30.80, *σ*_1_ = 0.6307.

Figure 5 shows the influence of the internal detuning parameter σ_1_ (i.e., the DC voltage) on the frequency response curves. It follows from Figure 5 that with the increase of σ_1_, the amplitude of the indirectly third mode decreases. The interval between the two Hopf bifurcation decreases until it vanishes.

**Figure 6:**
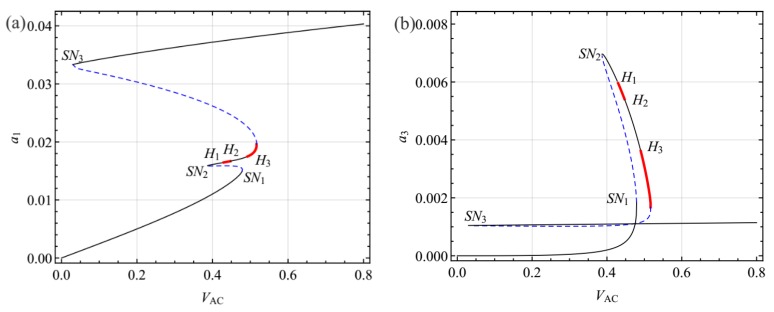
Force response curves for the first (**a**) and third (**b**) modes for *V*_DC_ = 23.80, *σ*_1_ = −0.8059, and *σ*_2_ = −0.3.

The variation of the response amplitude with excitation *V*_AC_ was examined when *σ*_1_ = −0.8059 and *σ*_2_ = −0.3. The force-response curves are shown in Figure 6. As the *V*_AC_ increases from 0, the amplitude of the response grows until a saddle node bifurcation occurs at *SN*_1_, resulting in a jump of the response to the other stable branch. As the *V*_AC_ increases further, the response loses stability via another saddle-node bifurcation at *SN*_3_. Increasing the *f* beyond *SN*_3_, the amplitude of the response for the first mode jumps up to another stable branch. To the left of this saddle node point, a second saddle node *SN*_2_ occurs. As *V*_AC_ decrease beyond *SN*_2_, the response loses stability via a Hopf bifurcation at *H*_1_ and regained stability via a Hopf bifurcation at *H*_2_. Then, this equilibrium solution encounters a Hopf bifurcation again at *H*_3_, resulting in the response losing stability and then jumping to the other larger stability branch with increasing *V*_AC_.

**Figure 7:**
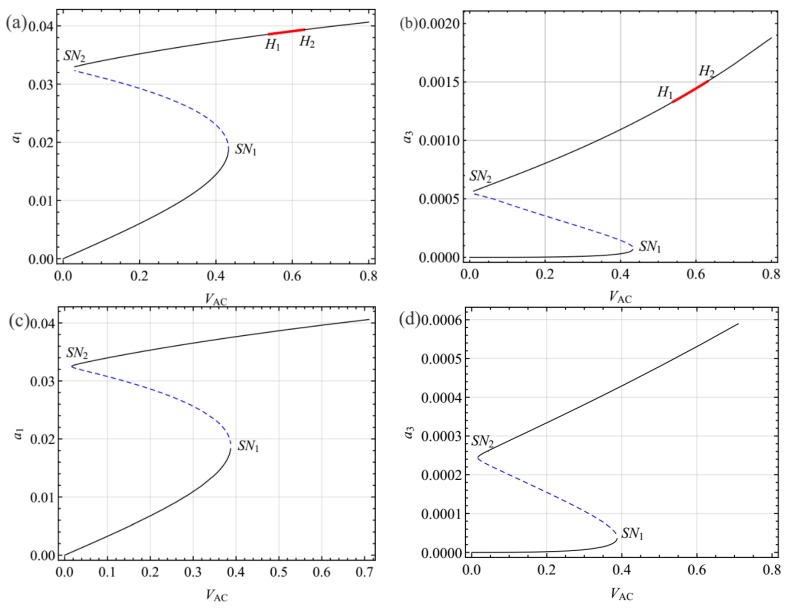
Variation of force response curves for various values of the internal detuning parameters for *σ*_2_ = −0.3: (**a**,**b**) *V*_DC_ = 28.00, *σ*_1_ = 0.005; (**c**,**d**) *V*_DC_ = 30.80, *σ*_1_ = 0.6307.

It may be noted that the response of the system has many Hopf bifurcations and saddle node bifurcations due to internal resonance. To investigate the effect of the internal resonance on the response, Figure 7 shows force response curves for *σ*_1_ = 0.005 and *σ*_1_ = 0.6307. As can be seen from Figure 7, as *σ*_1_ increases, Hopf bifurcation diminishes. When *σ*_1_ = 0.6307, i.e., *V*_DC_ = 30.80, the force response curves show behavior similar to the single-modal dynamics of the system.

## 5. Dynamic Solutions

When inspecting frequency responses and amplitude response curves, it can be found that many bifurcations may occur with variation of parameters. This calls for analysis of the dynamic response of the system. Hence, in the present section we explore the dynamic behavior caused by these bifurcations (especially the Hopf bifurcation points) using the numerical method. To this end, we employed the Galerkin scheme to discretize the governing equation into a set of nonlinear ordinary differential equations. We assumed the response of the MEMS arch in the form

(31) $$w\left(x,t\right)=w_{s}\left(x\right)+\upsilon \left(x,t\right)=w_{s}\left(x\right)+\sum_{i=1}^{N}\phi_{i}\left(x\right)q_{i}\left(t\right)$$

where *N* is the number of the modes retained in the discrete scheme, *ϕ_i_*(*x*) is the eigen function of the straight clamped-clamped beam, and *q_i_*(*t*) is the generalized coordinate of the transverse displacement. Substituting Equation (31) into Equation (3), using the Taylor expansion around the *w_s_*(*x*), multiplying the resultant equation by *ϕ_j_*(*x*), and then integrating the result over the domain yielded the second-order nonlinear ordinary differential equation

(32) $${\ddot{q}}_{i}+c{\dot{q}}_{i}+\sum_{j=1}^{N}K_{ij}^{\left(1\right)}q_{j}+\sum_{j=1}^{N}\sum_{m=1}^{N}K_{ijm}^{\left(2\right)}q_{j}q_{m}+\sum_{j=1}^{N}\sum_{m=1}^{N}\sum_{l=1}^{N}K_{ijml}^{\left(3\right)}q_{j}q_{m}q_{l}=F_{i}\cos\left(\Omega t\right)$$

where *i* = 1, 2, ..., *N*, and the overdot denotes the derivative with respective to time. $K_{ij}^{\left(1\right)}$, $K_{ijm}^{\left(2\right)}$, and $K_{ijml}^{\left(1\right)}$ are the linear, quadratic, and cubic stiffness coefficients, respectively. *F_i_* and Ω are the amplitude and frequency of the excitation, respectively. These coefficients have been included in Appendix B. Equation (32) can be numerically integrated using the Runge-Kutta technique to obtain the dynamic solution.

**Figure 8:**
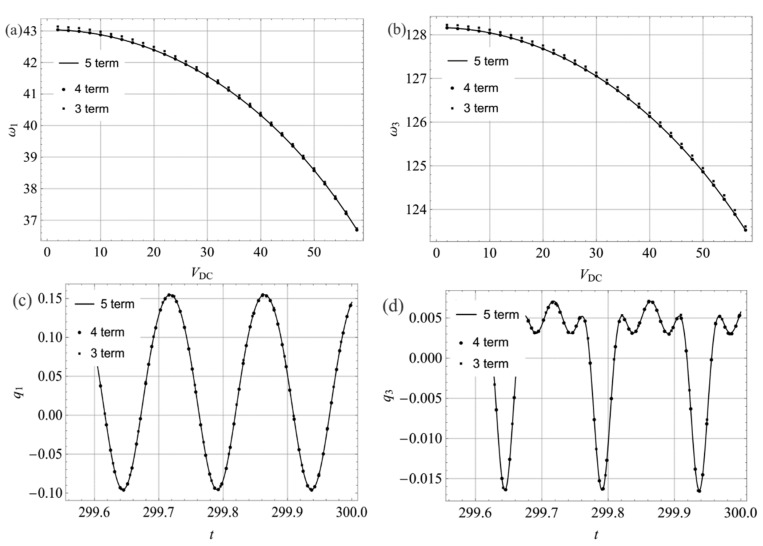
Discussion on the convergence of the Galerkin scheme: (**a**) the first natural frequency; (**b**) the second frequency; (**c**) time history of the first mode; (**d**) time history of the third mode.

For the Galerkin scheme, one key problem is its convergence. Figure 8a,b show the variation of the first two frequencies with *V_DC_* respectively, using three different values of *N*, namely, 3, 4, and 5. It should be noted that the first two frequencies are accurate enough when a 4-mode Galerkin truncation is employed. With regards to the parameters *c* = 0.01, *V*_DC_ = 23.80, and *V*_AC_ = 0.6, the response of the first two modes for the three different values of *N* are shown in Figure 8c,d, respectively. Similarly to the conclusion of natural frequency, the 4-mode Galerkin truncation is convergent. Hence, in the present paper, the dynamic solution of the system was solved by the 4-mode Galerkin scheme.

**Figure 9:**
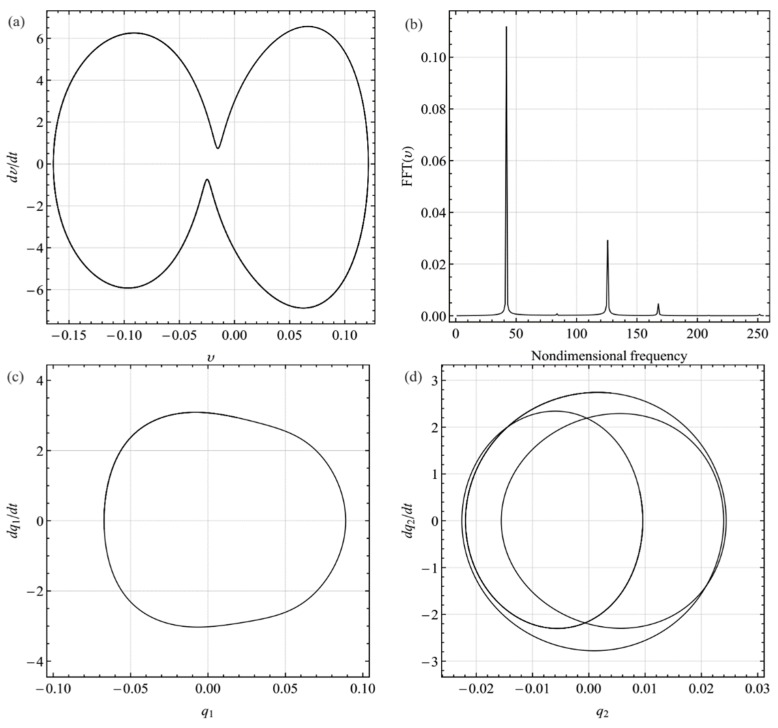
Dynamic response with internal resonance for the excitation frequency ratio Ω = 42.0812 (*σ*_2_ = −0.8): (**a**) phase-plane portrait; (**b**) Fourier spectrum; (**c**) projection onto $q_{1}^{{}^{\cdot }}$ − *q*_1_ plane; (**d**) projection onto $q_{2}^{{}^{\cdot }}$ − *q*_2_ plane. *q_i_* and $q_{i}^{{}^{\cdot }}$ (*i* = 1, 2) denote the generalized displacement and velocity of the *i*th mode, respectively.

To simulate dynamic behavior, we chose the same system parameters with Figure 4 and set the excitation frequency to 42.3627, i.e., *σ*_2_ = 0.55, in Figure 4. Figure 9a–d show the phase-plane portraits, Fourier spectrum, and projection of the response onto the phase plane of the first and third mode, respectively. It should be noted that the response contains components of the first and third modes, which indicates that the energy is transferred from the first mode to the third mode.

**Figure 10:**
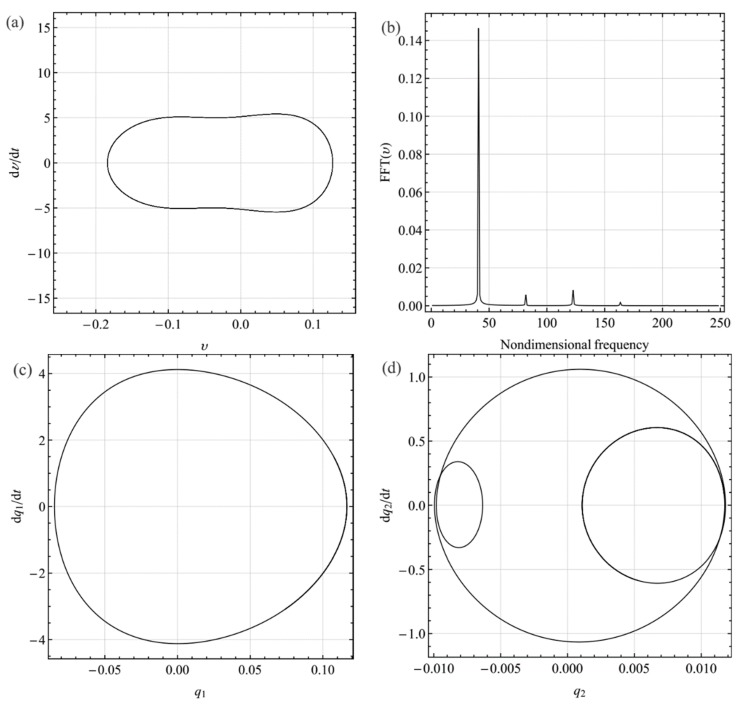
Dynamic response with internal resonance for the excitation frequency ratio Ω = 40.8506 (*σ*_2_ = −2.03069): (**a**) phase-plane portrait; (**b**) Fourier spectrum; (**c**) projection $q_{1}^{{}^{\cdot }}$ − *q*_1_ onto plane; (**d**) projection $q_{2}^{{}^{\cdot }}$ − *q*_2_ onto plane. *q_i_* and $q_{i}^{{}^{\cdot }}$ (*i* = 1, 2) denote the generalized displacement and velocity of the *i*th mode, respectively.

Decreasing the excitation frequency to 42.1127, which corresponds to a stable point at *σ*_2_ = −0.8 in Figure 4, Figure 10a–d show the phase-plane portraits, Fourier spectrum, and projection of the response onto the phase plane of the first and third mode, respectively. Compared with Figure 9, the response amplitude of the first mode in Figure 10 is larger. Also, the contribution of the third mode to the response is smaller.

**Figure 11:**
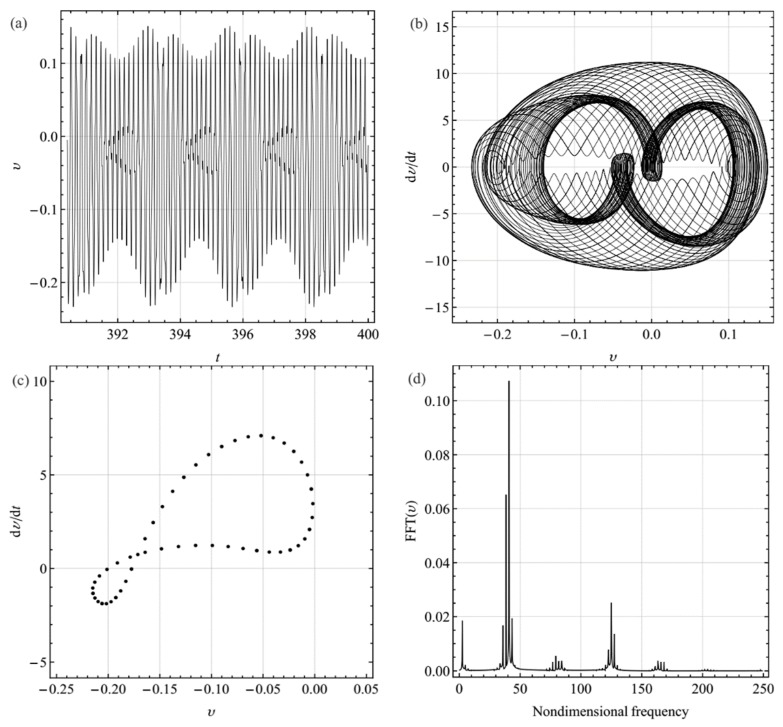
Quasi-periodic response for the excitation frequency ratio Ω = 40.8505 (*σ*_2_ = −2.0307): (**a**) time history; (**b**) phase-plane portrait; (**c**) Poincare sections; (**d**) Fourier spectrum.

Slightly decreasing the excitation frequency to 42.1027, which corresponds the Hopf point *H*_2_ at *σ*_2_ = −0.81, Figure 11a–d show the response of the system in terms of time histories, phase portraits, Fourier spectrum, and Poincare sections. It should be noted that the Fourier spectrum contains some other harmonic components and the Poincare sections present a closed loop. These phenomena indicate that the response is quasiperiodic. In addition, for the other values of the detuning parameter *σ*_2_ between *H*_1_ and *H*_2_ in Figure 4a,b, the response remains quasiperiodic and a similar behavior is obtained. Further, decreasing the excitation frequency to less than 40.6812, namely, *σ*_2_ = −2.2, the response shows a periodic response similar to Figure 10 again. To avoid repetition, these results have not been presented.

## 6. Conclusions

In this paper, the nonlinear response of a MEMS arch resonator with three-to-one internal resonance was investigated. Size effect and axial load were also considered. The multiple scales method was employed to solve the governing equation. The frequency and force response curves were obtained in the presence of three-to-one internal resonance. Results showed that even though the ratio between the first and second natural frequency of the MEMS arch can be three-to-one, the two modes cannot be coupled in the nonlinear sense and hence cannot exchange energy with each other. By contrast, the first and third mode can be coupled due to three-to-one internal resonance. Moreover, many nonlinear phenomena, such as softening spring behaviors, jumping, and Hopf bifurcation, can be detected due to the existence of internal resonance. Furthermore, the effect of different parameters on internal resonance was also studied. The results show that increasing the DC voltage weakens modal interaction due to internal resonance while decreasing the DC voltage level enhances modal interactions.

In addition, to simulate the dynamic solution of the system, a reduced-order model was derived by employing the Galerkin scheme. The dynamics of the MEMS arch were investigated reharding time history, phase-plane portrait, Poincare sections, and the Fourier spectrum. Periodic, quasiperiodic, and mixed responses were observed in the motion, which demonstrates the role of modal interactions due to internal resonance. The presented results can be used in the design and optimization of novel MEMS resonators and give insight into how modal interactions can affect the stability and the resonant responses of MEMS arch resonators.

## Appendix A

$$\begin{array}{ll} \chi_{1i}= & \alpha_{1}\phi_{i}^{\prime \prime }\left[3\int_{0}^{1}{\phi^{\prime }}_{i}^{2}dx+\int_{0}^{1}w_{s}^{\prime }\left(2\psi_{1i}^{\prime }+4\psi_{2i}^{\prime }\right)dx\right]+\alpha_{1}w_{s}^{\prime \prime }\int_{0}^{1}\phi_{i}^{\prime }\left(2\psi_{1i}^{\prime }+4\psi_{2i}^{\prime }\right)dx+2\alpha_{1}\left(\psi_{1i}^{\prime \prime }+2\psi_{2i}^{\prime \prime }\right)\int_{0}^{1}w_{s}^{\prime }\phi_{i}^{\prime }dx\\ & +\frac{12\alpha_{2}V_{DC}^{2}}{{\left(1+w_{s}\right)}^{5}}\phi_{i}^{3}-\frac{6\alpha_{2}V_{DC}^{2}}{{\left(1+w_{s}\right)}^{4}}\phi_{i}\left(\psi_{1i}+\psi_{2i}\right)\;i=1,3 \end{array}$$

$$\begin{array}{ll} \chi_{ij}= & \alpha_{1}\phi_{j}^{\prime \prime }\left[4\int_{0}^{1}\phi_{i}^{\prime }\phi_{j}^{\prime }dx+2\int_{0}^{1}w_{s}^{\prime }\left(\psi_{3}^{\prime }+\psi_{4}^{\prime }\right)dx\right]+\alpha_{1}\phi_{i}^{\prime \prime }\left[2\int_{0}^{1}\phi_{j}^{\prime }\phi_{j}^{\prime }dx+4\int_{0}^{1}w_{s}^{\prime }\psi_{2j}^{\prime }dx\right]\\ & +\alpha_{1}w_{s}^{\prime \prime }\left[2\int_{0}^{1}\phi_{j}^{\prime }\left(\psi_{3}^{\prime }+\psi_{4}^{\prime }\right)dx+4\int_{0}^{1}\phi_{i}^{\prime }\psi_{2j}^{\prime }dx\right]+2\alpha_{1}\left(\psi_{3}^{\prime \prime }+2\psi_{4}^{\prime \prime }\right)\int_{0}^{1}w_{s}^{\prime }\phi_{j}^{\prime }dx\\ & +\frac{24\alpha_{2}V_{DC}^{2}}{{\left(1+w_{s}\right)}^{5}}\phi_{i}\phi_{j}^{2}-\frac{6\alpha_{2}V_{DC}^{2}}{{\left(1+w_{s}\right)}^{4}}\left(\psi_{3}\phi_{j}+\psi_{4}\phi_{j}+2\psi_{2j}\phi_{i}\right)\;i,j=1,3\;\mathrm{and}\;i\neq j \end{array}$$

$$\begin{array}{ll} \chi_{5}= & \alpha_{1}\phi_{1}^{\prime \prime }\left[3\int_{0}^{1}{\phi^{\prime }}_{1}^{2}dx+\int_{0}^{1}w_{s}^{\prime }\psi_{11}^{\prime }dx\right]+2\alpha_{1}w_{s}^{\prime \prime }\int_{0}^{1}\phi_{1}^{\prime }\psi_{11}^{\prime }dx+2\alpha_{1}\psi_{11}^{\prime \prime }\int_{0}^{1}w_{s}^{\prime }\phi_{1}^{\prime }dx\\ & +\frac{4\alpha_{2}V_{DC}^{2}}{{\left(1+w_{s}\right)}^{5}}\phi_{1}^{3}-\frac{6\alpha_{2}V_{DC}^{2}}{{\left(1+w_{s}\right)}^{4}}\phi_{1}\psi_{11} \end{array}$$

$$\begin{array}{ll} \chi_{6}= & 2\alpha_{1}\phi_{1}^{\prime \prime }\left(\int_{0}^{1}\phi_{1}^{\prime }\phi_{3}^{\prime }dx+\int_{0}^{1}w_{s}^{\prime }\psi_{4}^{\prime }dx\right)+\alpha_{1}\phi_{3}^{\prime \prime }\left(\int_{0}^{1}\phi_{1}^{\prime }\phi_{1}^{\prime }dx+2\int_{0}^{1}w_{s}^{\prime }\psi_{11}^{\prime }dx\right)+2\alpha_{1}w_{s}^{\prime \prime }\int_{0}^{1}\left(\phi_{3}^{\prime }\psi_{11}^{\prime }+\phi_{1}^{\prime }\psi_{4}^{\prime }\right)dx\\ & +2\alpha_{1}\psi_{4}^{\prime \prime }\int_{0}^{1}w_{s}^{\prime }\phi_{1}^{\prime }dx+2\alpha_{1}\psi_{11}^{\prime \prime }\int_{0}^{1}w_{s}^{\prime }\phi_{3}^{\prime }dx+\frac{12\alpha_{2}V_{DC}^{2}}{{\left(1+w_{s}\right)}^{5}}\phi_{3}\phi_{1}^{2}-\frac{6\alpha_{2}V_{DC}^{2}}{{\left(1+w_{s}\right)}^{4}}\left(\phi_{1}\psi_{4}+\psi_{11}\phi_{3}\right) \end{array}$$

$$F\left(x\right)=-\frac{\alpha_{2}V_{DC}V_{AC}\cos\left(\Omega t\right)}{{\left(1+w_{s}\right)}^{2}}$$

$$f_{1}=\frac{1}{\omega_{1}}\int_{0}^{1}F\left(x\right)\phi_{1}\left(x\right)dx\;S_{kk}=\frac{8}{\omega_{k}}\int_{0}^{1}\chi_{1k}\left(x\right)\phi_{k}\left(x\right)dx$$

$$S_{jk}=\frac{8}{\omega_{j}}\int_{0}^{1}\chi_{jk}\left(x\right)\phi_{j}\left(x\right)dx\;j,k=1,3\;\mathrm{and}\;j\neq k$$

$$\Lambda_{1}=\frac{8}{\omega_{1}}\int_{0}^{1}\chi_{5}\left(x\right)\phi_{3}\left(x\right)dx\;\Lambda_{3}=\frac{8}{\omega_{3}}\int_{0}^{1}\chi_{6}\left(x\right)\phi_{1}\left(x\right)dx$$

## Appendix B

$$\begin{array}{l} K_{ij}^{\left(1\right)}=\int_{0}^{1}\left(k_{1}\phi_{i}^{iv}+\lambda^{2}\phi_{i}^{\prime \prime }\right)\phi_{j}dx-2\alpha_{1}\int_{0}^{1}w_{s}^{\prime \prime }\phi_{j}dx\int_{0}^{1}w_{s}^{\prime }\phi_{i}^{\prime }dx-2\alpha_{2}V_{DC}^{2}\int_{0}^{1}\frac{\phi_{i}\phi_{j}}{{\left(1+w_{s}\right)}^{3}}dx\\ K_{ij}^{\left(2\right)}=-\alpha_{1}\int_{0}^{1}w_{s}^{\prime \prime }\phi_{j}\mathrm{dx}\int_{0}^{1}\phi_{i}^{\prime }\phi_{m}^{\prime }dx-2\alpha_{1}\int_{0}^{1}w_{s}^{\prime }\phi_{m}^{\prime }dx\int_{0}^{1}\phi_{i}^{\prime \prime }\phi_{j}dx+3\alpha_{2}V_{DC}^{2}\int_{0}^{1}\frac{\phi_{i}\phi_{j}\phi_{m}}{{\left(1+w_{s}\right)}^{4}}dx\\ K_{ij}^{\left(3\right)}=-\alpha_{1}\int_{0}^{1}\phi_{i}^{\prime \prime }\phi_{j}dx\int_{0}^{1}\phi_{l}^{\prime }\phi_{m}^{\prime }dx-4\alpha_{2}V_{DC}^{2}\int_{0}^{1}\frac{\phi_{i}\phi_{j}\phi_{m}\phi_{n}}{{\left(1+w_{s}\right)}^{5}}dx\\ F_{i}=-2\alpha_{2}V_{DC}V_{AC}\int_{0}^{1}\frac{\phi_{j}}{{\left(1+w_{s}\right)}^{2}}dx \end{array}$$
